# Phylogenetic Diversity of the Red Swamp Crayfish *Procambarus clarkii* and Its Dispersal Pattern in Northern and Central Italy

**DOI:** 10.3390/biology12020313

**Published:** 2023-02-15

**Authors:** Melissa Scoparo, Irene Cardinali, Gianandrea La Porta, Barbara Caldaroni, Gabriele Magara, Ambrosius Josef Martin Dörr, Antonia Concetta Elia, Hovirag Lancioni

**Affiliations:** Department of Chemistry, Biology and Biotechnology, Università degli Studi di Perugia, 06123 Perugia, Italy

**Keywords:** Cambaridae, dispersal routes, invasive species, mitochondrial markers, molecular phylogeny, multiple introductions

## Abstract

**Simple Summary:**

The red swamp crayfish *Procambarus clarkii* is one of the most threatening freshwater species in the world. Since 2016, it has been included in the Invasive Alien Species list of the European Union, and it is still actively colonizing new territories at the expense of native crayfish. The present study aimed to provide a genetic characterization for a better understanding of the invasion routes of this alien species in the Italian Peninsula. The analysis of the mitochondrial DNA of 153 *P. clarkii* samples collected from five Italian lakes and one river showed the presence of unique maternal lines in four of these basins, thus confirming the hypothesis of multiple introductions and strengthening the idea that knowledge of dispersion routes can be very useful to predict the invasiveness of this species and create control strategies to preserve biodiversity.

**Abstract:**

The red swamp crayfish *Procambarus clarkii* is one of the most threatening freshwater species in the world. The aim of this study is to provide a better understanding of the phylogeography and the invasion routes of *P. clarkii* populations in the Italian Peninsula through the analysis of mitochondrial phylogeny. Mitochondrial control region and cytochrome c oxidase subunit I (COI) sequences of 153 samples collected from six Italian basins were analyzed and compared to worldwide data. Except for the lakes Bolsena and Posta Fibreno, a high genetic variability was found in the other basins. The mitochondrial DNA pattern of *P. clarkii* from the lakes Candia and Massaciuccoli confirmed the hypothesis of double introduction events. Another entry point could be represented by Lake Trasimeno, which shows haplotypes originating from Louisiana and not shared with other Italian basins. Moreover, unique lineages were also found in the Stella River, thus enhancing the hypothesis that multiple introductions of *P. clarkii* occurred in northern and Central Italy and strengthening the idea that knowledge about the dispersion routes of this alien species can be useful to predict its invasiveness and elaborate control strategies to preserve biodiversity.

## 1. Introduction

Biological invasions are believed to be one of the main drivers of global change, with negative environmental [1] and socio-economic impacts [2], as the human-mediated transfer of species to areas different from their native geographic ranges can cause significant changes to the recipient ecosystems and their biodiversity.

The Louisiana red swamp crayfish *Procambarus clarkii* (Girard, 1852) Cambaridae has spread from its native range (north-eastern Mexico and south-central USA) through both accidental and intentional human actions, becoming a worldwide invader, except for Australia and Antarctica [3,4]. The high rate of invasiveness is due to its connotation as an r-strategist species, which is characterized by rapid growth, precocious sexual maturity and high fertility [5]. Furthermore, this crayfish can survive at different degrees of dehydration, selecting moist environments [6], and can even tolerate a salt concentration of 35‰ [7] and environmental contaminants [8,9].

It is an asymptomatic potential carrier of the crayfish plague (*Aphanomyces astaci*) and an importer of pathogenic microfungal taxa such as *Phoma glomerata* (ubiquitous Coelomycetes), potentially dangerous to human health, flora and fauna [10,11]. 

Due to the abovementioned characteristics, *P. clarkii* is reported among the 100 worst invasive alien species (IAS) in the Delivering Alien Invasive Species Inventory for Europe (DAISIE) in Regulation (EU) No. 1143/2014 of the European Parliament, and in the Council of 22 October 2014 on the prevention and management of the introduction and spread of invasive alien species. Invasive alien species management is closely related to the identification of the invasion routes and could be very helpful to establish possible approaches to prevent and control alien species invasions [12].

In this scenario, population genetics represents an informative tool to facilitate the effective ecological restoration of invaded ecosystems [13], and to reconstruct the phylogeny, trace possible invasion paths and estimate the frequency of introductions [14]. The successful invasion of an invasive species into a new environment might cause an increase in genetic diversity, because of its multiple introductions, hybridization and adaptation [15,16]. The invasion process determines a genetic diversity for this invasive species, pointing out the importance of ecological and anthropogenic factors in shaping its variability [16]. Mitochondrial DNA (mtDNA) can be particularly useful in tracing ancestral relationships among individuals because of maternal inheritance and the high mutation rate [17]. In particular, the region of COI has a considerable potential for the DNA barcoding identification system for crustaceans [18], to identify individuals at species level and to replace the current methods used in the official control of foodstuffs [19], while control region (CR), the non-coding and hypervariable area of the mtDNA that evolves three to five times more rapidly than the rest [20], represents a powerful molecular marker for phylogenetic and phylogeographic studies. The global invasion history of *P. clarkii* was reconstructed by analyzing the COI gene from 1416 samples, reporting two independent invasion routes in the USA, more introduction routes towards Europe and a single event into Asia [21]. Nevertheless, most genetic studies of *P. clarkii* were carried out in Chinese populations and highlighted a high level of genetic diversity, with irregularly distributed populations in accordance with the genetic differentiation as a consequence of the geographic isolation [22,23,24,25,26].

In 2014, Paulson and Martin analyzed the genetic landscape of *P. clarkii* in Ash Meadows (Nevada), demonstrating how measures of genetic distance and genetic diversity within populations could be very helpful to infer the frequency and the location of dispersal routes used by an invasive species [13]. A survey of genetic variation in the Santa Monica Mountains (South California) was performed by Quan and colleagues [27], who identified a source population and determined whether multiple introductions had occurred in this area. The mtDNA COI analysis of North-American Cambaridae and European Astacidae showed a close relationship between these two populations [28], while different levels of intraspecific mtDNA variation in the European continent were reported [29]. 

The invasion of *P. clarkii* in Europe was firstly recorded in 1973, with the deliberate and legal introduction of this species into Spain as a commercial resource. The crayfish was firstly imported from New Orleans (LA, USA) into a farm in the Spanish province of Badajoz (Habsburg–Lorraine, 1986) and then into a pond near Guadalquivir (Puebla del Rıo, Seville) [30]; then, it almost invaded all the European continent.

Several studies focused on its genetic diversity, and a low variability was observed both in France [31] and in the Iberian Peninsula [32]. *Procambarus clarkii* was introduced in Italy in 1977, even if its presence was recorded for the first time in 1989. The existence of *P. clarkii* in this area could be due to the presence of a small intensive crayfish farm from 1977 to 1985. It is not clear whether the release occurred accidentally or if it was human-mediated [33]. The species colonized a stream in the hydrogeographic basin of the Benna torrent, a tributary of the Po River, precisely in the Carmagnola area (Turin province; northern Italy). Nevertheless, to date there are few available data for the *P. clarkii* mitochondrial control region, which have allowed a better understanding of its rapid invasion in China [34] and an identification of typical genetic variants in the Italian biotope of Lake Trasimeno (Central Italy) [35]. In the present study, we analyzed the genetic variation of the mtDNA control region and cytochrome c oxidase subunit I (COI) in six *P. clarkii* populations of northern and Central Italy, in order to (i) outline their genetic relationships, (ii) investigate their origins and dispersal routes, (iii) identify likely source populations and (iv) determine whether multiple introductions have occurred in these areas, by focusing on the role of single versus multiple events.

## 2. Materials and Methods

### 2.1. Sampling and Study Area

A total of 153 individuals of *P. clarkii* were collected from six Italian basins with different biotic and abiotic characteristics: five lakes (Bolsena, Candia, Massaciuccoli, Posta Fibreno and Trasimeno) and one river (Stella).

Lake Candia (ZSC-IT1110036) is in the north-west of the Italian Peninsula and is located near to Carmagnola (Turin province), which is considered the first entry point of this invasive species in Italy. Lake Massaciuccoli (ZSC-IT5120021) is a brackish lake located in the Migliarino, San Rossore, Massaciuccoli nature park (Lucca province, Central Italy) and is supposed to be the second biotope invaded by *P. clarkii* [36], which then spread throughout both the whole peninsula and Sicily and Sardinia [37,38]. The typical ecosystem conditions of Lake Trasimeno (ZSC-IT5210018), eutrophic and shallow, have been extremely favorable for the maintenance of the species, which is well acclimatized, revealing a stable population structure during the last two decades [35]. The volcanic Lake Bolsena (ZSC-IT5210018) (Central Italy) is located within the Vulsini Volcanic district, which marks the north-western end of the Roman Province, while Lake Posta Fibreno (ZSC-IT6050015) (Central Italy, Latium district) is a spring lake [39] whose recharge area is the karstic environment of the Apennines. The Stella River is in north-east of Italy, and was chosen to extend our dataset even to this side of the peninsula, because of its distinctive biotic and abiotic features. The sampling site was near Precenicco (Udine province), located in the final stretch of the Stella River. This area is a meander connected from an ecological point of view to the Natura 2000 site, which affects the entire lagoon (ZSC-IT3320036).

### 2.2. Genetic Analyses

All samples were analyzed in order to determine DNA content and to sequence two mtDNA markers: the control region (CR) and the cytochrome c oxidase subunit I (COI).

Genomic DNA was extracted both from muscle and hepatopancreas tissues using the Wizard^®^ Genomic DNA Purification Kit (Promega Corporation, Madison, WI, USA) and DNeasy^®^ Tissue Kit di Qiagen (Hilden, Germany, EU) and a good quality of DNA was obtained from both.

The primers used for mtDNA control-region amplification were previously published [34], and the reaction conditions were as follows: initial denaturation at 95 °C for 2 min, followed by 35 cycles of denaturation at 95 °C for 30 s, annealing at 55 °C for 1 min and elongation at 72 °C for 1 min, followed by final extension at 72 °C for 10 min. Besides, a fragment of the mitochondrial gene coding for the cytochrome c oxidase subunit I was amplified using the primers published by Folmer et al., 1994 [40].

The PCR reactions contained 1X Buffer GoTaq, 2.5 mM of each deoxyribonucleotide triphosphate (dNTP), 0.3 µM of each primer, 0.03 U/µL of GoTaq DNA polymerase (Promega Corporation; Madison, WI, USA), 30 ng of genomic DNA and H_2_O to a final volume of 25 µL. The PCR amplification was carried out as follows: 94 °C for 2 min, followed by 35 cycles of 95 °C for 30 s, 55 °C for 1 min, 72 °C for 1 min and then 72 °C for 10 min. The PCR fragments were purified using exonuclease I and alkaline phosphatase (ExoSAP-IT enzymatic system-USB Corporation, Cleveland, OH, USA) and subsequently Sanger-sequenced with the following primers: LCO1490 and HCO2198 [40] for COI sequencing and a specifically designed primer (5′-CTTCTAAAAATGTTCCCCCC-3′) for CR region, by using Primer3 software. Sequences were aligned to the sequence considered as reference mitogenome (JN991197, [41]) for the haplotype (HT) annotation through the Sequencher software (www.genecodes.com (accessed on 12 August 2022)). In order to ensure the uniformity of all sequences, they were trimmed at the same range, from the nucleotide position (np) 4717 to np 5434 for the mtDNA CR marker, and from np 78 to np 651 for the COI region.

The COI region was investigated in 53 samples, selecting at least 2 samples for each CR HT from each basin.

Mitochondrial DNA sequence variation parameters were estimated by using DnaSP 5.1 software (www.ub.edu/dnasp/index_v5.html (accessed on 12 August 2022)). Haplotype number codes were assigned to each sample as previously classified in Dörr et al., 2021 [35] and with increasing numbering only for our convenience.

The evolutionary relationships among the haplotypes here identified were evaluated for each single mtDNA marker through median-joining trees built using Network software v.10.2.

In order to graphically display and summarize the mitogenetic relationships among the analyzed populations, a Principal Component Analysis (PCA) based on concatenated mtDNA (COI and CR) haplotypes was performed using the Excel software implemented by XLSTAT. Haplotype frequencies were used as input data.

Finally, to construct a Bayesian phylogeny for the two combined mtDNA regions, we used BEAST.v2.7.3 and the analyses were run for 100,000,000 generations [42].

## 3. Results and Discussion

The migratory and diffusion routes of *P. clarkii* have been widely described. It is a species native to Louisiana and arrived in Europe with a first translocation into Spain in 1973 [36]. Its ecological adaptability allows it to colonize and survive in different types of basins, thus causing the decline of many autochthonous species.

Here, we analyzed for the first time two mtDNA markers (the control region and cytochrome c oxidase subunit I) of *P. clarkii*, sampled in six Italian basins (Table S1) and compared them with worldwide published data. In general, most available sequences are from the COI gene, even though the hypervariable regions of mtDNA CR seem to have achieved better results [20].

### 3.1. Genetic Analysis of Mitochondrial DNA Control Region

The overall alignment of 718 base pairs (from nucleotide position (np) 4717 to np 5434) of the 153 control-region sequences from this study showed an overall low haplotype diversity (Hd = 0.726), with a total of ten haplotypes (HTs) and 22 polymorphic sites (S) detected (Table 1).

We observed low values of haplotype diversity for individuals from lakes Bolsena and Trasimeno, and the Stella River (Hd values of 0.431, 0.408 and 0.400, respectively), whereas the highest value was recorded for Lake Massaciuccoli (Hd = 0.806). These results seem to confirm the hypothesis that considers Lake Massaciuccoli as the area from which this invasive species spread across the Italian Peninsula through different man-made translocations [36,43].

**Figure 1:**
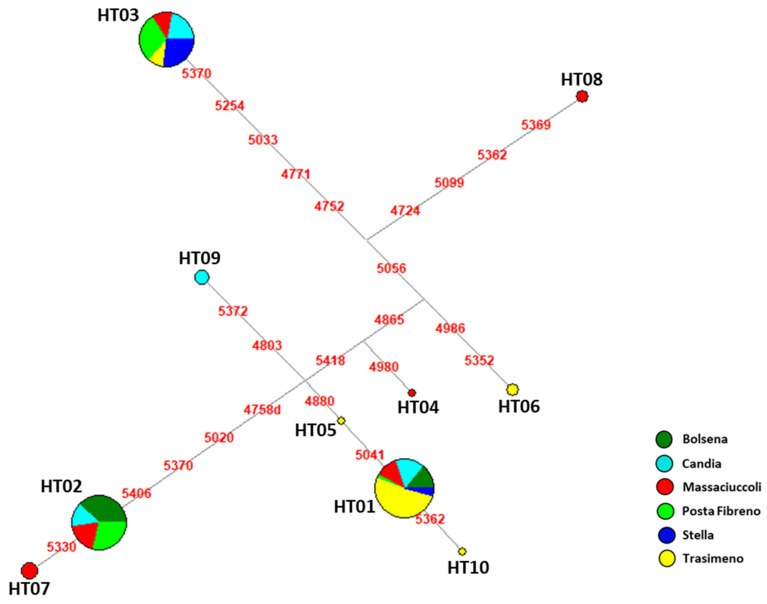
Median-Joining Network based on 153 control-region sequences (nps 4717–5434) belonging to six *P. clarkii* populations from Italy. Circles represent the haplotypes and are proportional to the observed frequency in 153 samples (Table S2), while colors indicate the different basins: Bolsena (dark green), Candia (light blue), Massaciuccoli (red), Posta Fibreno (light green), Stella River (blue) and Trasimeno (yellow).

Among ten mtDNA control-region haplotypes, the most represented was HT01, present in all Italian basins analyzed, followed by HT02 and HT03, with frequencies of 0.34, 0.29 and 0.27, respectively (Table S2). The other HTs (HT04-HT10) were found each in only one basin with frequencies spanning from 0.01 to 0.03. The network analysis showed an interesting genetic variability above all in Lake Massaciuccoli and Lake Trasimeno, with each one presenting three unique haplotypes (HT04, HT07 and HT08; HT05, HT06 and HT10, respectively) (Figure 1).

The comparison with available sequences from USA, China and Japan [34], uniformed to our range, showed only three haplotypes (HT02, HT09 and HT10) shared with five samples from Louisiana (Tables S3 and S4). Among them, HT10 is one of the four ancestral haplotypes identified by Li and colleagues [34] and is present only in Lake Trasimeno, thus highlighting that some *P. clarkii* individuals could have arrived in this basin through a direct translocation, probably from Spain, and not through a dispersal event from Massaciuccoli.

Notwithstanding the general low genetic diversity, all the Italian populations showed haplotypes never observed before, with one mtDNA lineage (HT01) present in all basins here analyzed, but seven unique haplotypes (HT04-HT10) retrieved in three lakes (Candia, Massaciuccoli and Trasimeno), supporting the hypothesis of multiple introductions from different areas [21]. In particular, Lake Candia is near to the presumed initial entry point in Italy [33], and Massaciuccoli Park is considered the second invaded biotope [36], while the typical Trasimeno Lake revealed a stable population structure during the last two decades [35].

### 3.2. Genetic Analysis of Mitochondrial Cytochrome Oxidase Subunit I

The analysis of 53 COI sequences showed a very low variability, with an overall haplotype diversity of 0.549 and a total of six haplotypes (HTCOI01-HTCOI06) identified (Table 2).

**Figure 2:**
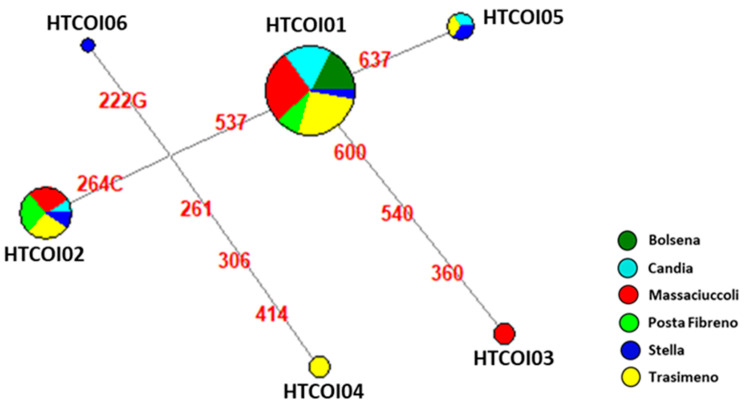
Median-Joining Network based on 53 COI sequences (nps 78–651) belonging to six *P. clarkii* populations from Italy. Circles represent the haplotypes and are proportional to the observed frequency in 53 samples (Table S2), while colors indicate the different basins: Bolsena (dark green), Candia (light blue), Massaciuccoli (red), Posta Fibreno (light green), Stella River (blue) and Trasimeno (yellow).

Despite the low number of analyzed samples from the Stella River, this basin appears to be the most heterogeneous, with four COI haplotypes identified (HTCOI01, HTCOI02, HTCOI05 and HTCOI06) (Figure 2; Table S2). The opposite results were found for Lake Bolsena, which showed only the most frequent haplotype (HTCOI01) among our samples and recorded in all six basins (Figure 2; Table S2).

By looking at the network analysis, there are three unique haplotypes (HTCOI03, HTCOI04 and HTCOI06) found in lakes Trasimeno and Massaciuccoli and the Stella River, respectively. The comparison with published sequences pinpointed a worldwide distribution of HTCOI01, found in American [13,27,44], European [28,29,31] and Chinese [22,23,25,26] samples (Table S3). In particular, HTCOI01 and HTCOI04 seem to be present at high frequencies in the native range of Eastern Louisiana, while HTCOI03 predominate in Western Louisiana [21]. *Procambarus clarkii* shows a defined spatial genetic structure within the Iberian Peninsula, caused by two different invasion events that originated one genetic cluster in Portugal and another in Spain [16]. After the translocation into the Iberian Peninsula, the presence of HTCOI04 and HTCOI06 only in Trasimeno and Massaciuccoli, respectively, could testify for independent invasions towards the Italian Peninsula, while concerning HTCOI06, found above all in Portugal [21], it was retrieved only in the Stella River among our samples, thus suggesting a direct connection between these two areas.

### 3.3. Worldwide Genetic Variability and Phylogeny

**Figure 3:**
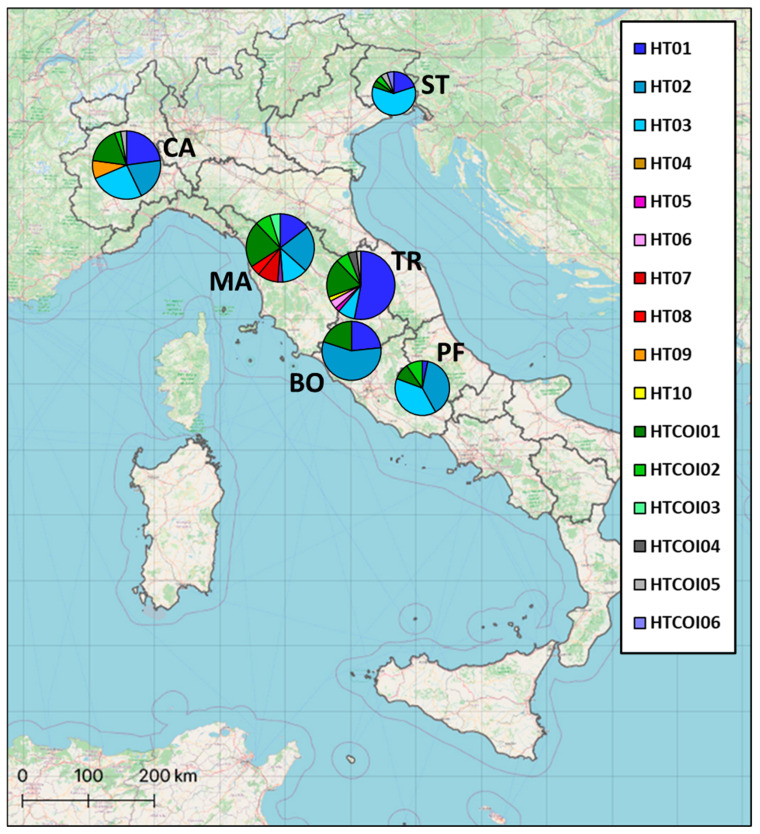
Sampling locations and frequency distribution of mtDNA haplotypes from 153 control-region and 53 COI sequences belonging to six Italian *P. clarkii* populations. Basin codes as in Table 1.

By considering both mitochondrial markers (CR and COI), our analysis highlighted four heterogeneous *P. clarkii* populations (the Candia, Massaciuccoli, Stella and Trasimeno basins), each presenting different unique haplotypes among our samples, in some cases shared with published data from other countries (Figure 3; Table S4).

Even if in the invaded areas a decline in diversity could be observed due to a founder effect, most studies reported high levels of genetic variability within and among introduced populations from non-native ranges [45]. This is also evident in this study, as the high genetic variation observed in the Italian Peninsula suggests that multiple independent invasions occurred into the region.

Moreover, through an accurate comparison between our CR and COI haplotypes, we observed that in some cases different CR HTs correspond to different COI HTs, while in other cases we found a direct connection, thus enhancing the importance of investigating different molecular markers in parallel. Control-region HT06 appears paired to HTCOI04, HT08 to HTCOI03 and HT09 to HTCOI01. While HTCOI01 is the most common COI haplotype among our samples and is connected to almost all CR haplotypes here identified, HTCOI03, typical of Lake Massaciuccoli, and HTCOI04, exclusively found in Lake Trasimeno, are each related to only one CR haplotype (HT08 and HT06) and have been stated as originating from western and eastern Louisiana, respectively [21]; this finding could confirm almost two distinct and direct introductions into Central Italy. Another noteworthy result concerns HTCOI02, which was found in almost all basins together with HT03, except for one sample from Massaciuccoli, which presents HT07, indicating two different source populations.

**Figure 4:**
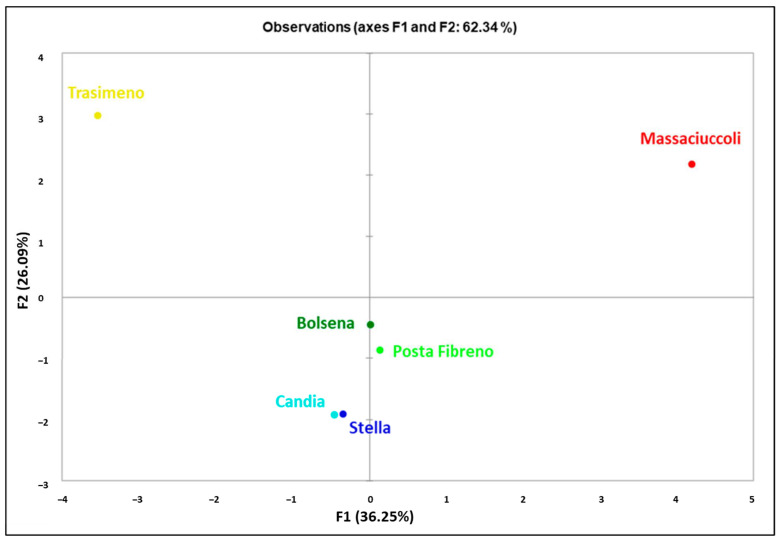
A two-dimensional basin-based bi-plot of concatenated mtDNA haplotype profiles (COI and CR) from the six Italian populations of *P. clarkii* here analyzed.

The analysis of haplotypes obtained from combined mtDNA markers (Table S5) showed a geographic differentiation in the PCA representing the mitochondrial genetic landscape in North–Central Italy (Figure 4).

The results derived from the merged dataset highlighted northern sites (Candia and Stella) clustering together, as for lakes Bolsena and Posta Fibreno, which showed a similar maternal clustering. Conversely, *P. clarkii* sampled in the Massaciuccoli and Trasimeno sites are clearly separated in the first and second quarter from the PC2, which pushes these two populations apart from each other due to the different distribution of concatenated haplotypes.

**Figure 5:**
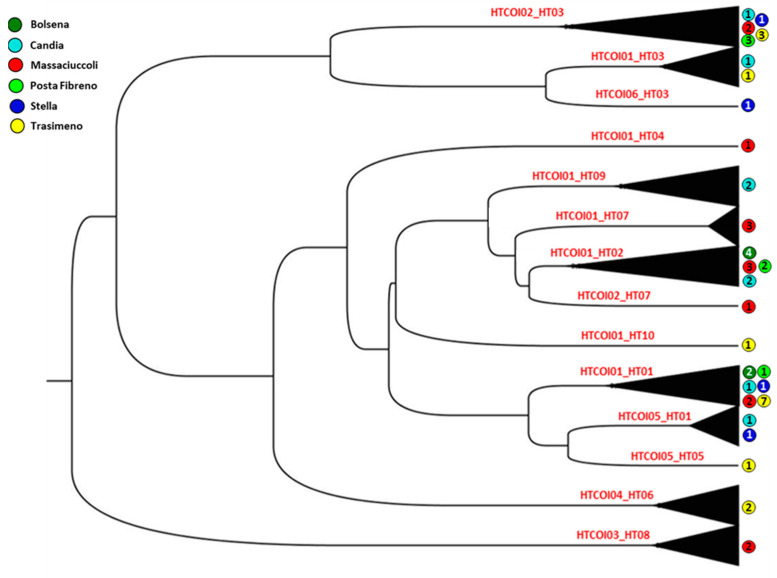
Bayesian phylogenetic tree based on concatenated mtDNA (COI and CR) haplotypes of six *P. clarkii* populations. Number of samples from the relative site is reported in each circle. Haplotype codes as in Table S5.

Then, the Bayesian phylogeny generated from the 53 samples analyzed for both mitochondrial (COI and CR) markers showed 14 combined haplotypes and pointed out the presence of unique haplotypes above all in the Massaciuccoli and Trasimeno lakes, thus confirming our results and the possibility of two distinct introductions of *P. clarkii* into Central Italy (Figure 5).

**Figure 6:**
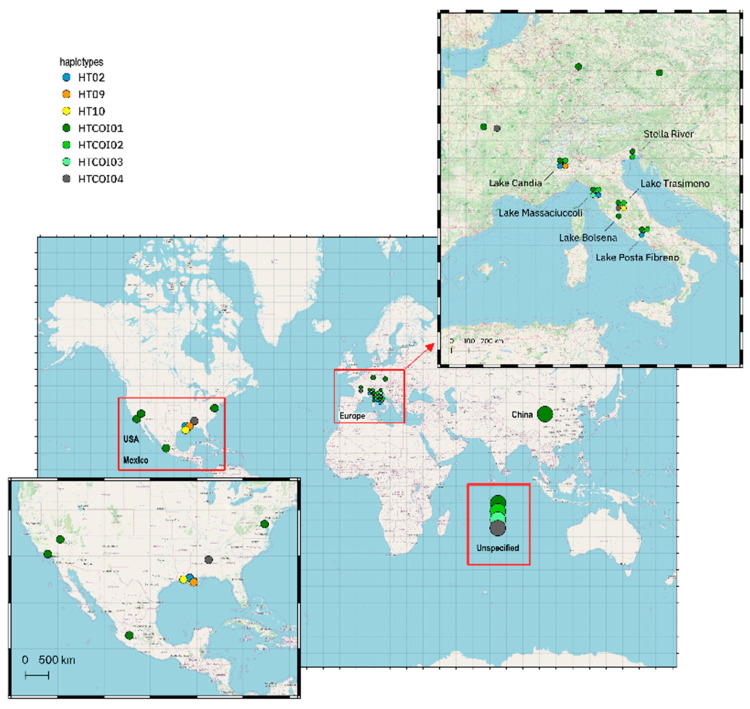
Geographic distribution of mtDNA haplotypes (mitochondrial control region and COI) from this study observed in other countries. Further details are reported in Tables S1 and S3.

In a worldwide context, the analysis of mtDNA CR and COI haplotypes here identified highlighted the highest genetic variability in the USA with respect to the other geographic areas previously described (Figure 6).

Even though there are only limited data we could consider for a comparison with our results, due to differences in type and length between our data and those previously published, this scenario is in agreement with the literature, as we could observe the maximum genetic variation within the native range of *P. clarkii*. Among the haplotypes identified in this study (HT01-HT10 and HTCOI01-HTCOI06), only seven (three from CR and four from COI) were retrieved in other countries. Except for those samples without a specified geographic origin (here called unspecified), the other areas showed a decline in mtDNA diversity, as stated for the invaded regions. The control-region haplotypes HT02, HT09 and HT10 were previously found only in Louisiana [34] and were not detected in samples from China, where only HTCOI01 was reported [22,23,25,26].

## 4. Conclusions

The comparative analysis between our findings and those retrieved from GenBank could be improved by increasing the number of samples and sampling sites and expanding the molecular analysis at higher levels in order to complete the COI sequence or analyze the complete mitogenome. Nevertheless, the results here described demonstrate the importance of the mitochondrial genome (especially its control region) as a valuable molecular marker for exploring crayfish phylogeography. Additionally, our genetic data confirmed the hypothesis of the double introduction events of *P. clarkii* in the north-western part of the Italian Peninsula (specifically shown by the genetic variability of the Candia and Massaciuccoli basins), and we here propose another entry point represented by Lake Trasimeno, which shows unique haplotypes originating from Louisiana and not shared with the other Italian basins here analyzed. Lastly, but no less interesting, the presence of unique haplotypes of *P. clarkii* also in the Stella River could enhance the hypothesis of multiple invasion routes into the Italian Peninsula, which deserves to be further investigated.

## Figures and Tables

**Table 1:** Estimates of mtDNA control-region genetic diversity indexes for all basins here analyzed (N = number of samples; π = nucleotide diversity; Nh = number of haplotypes; Hd = haplotype diversity; S = number of polymorphic sites; k = Tajima’s average number of nucleotide differences).

Basin	Basin Code	N	π	Nh	Hd	S	k
Bolsena	BO	24	0.003	2	0.431	5	2.156
Candia	CA	27	0.008	4	0.749	14	5.806
Massaciuccoli	MA	27	0.008	6	0.806	18	5.607
Posta Fibreno	PF	25	0.007	3	0.560	12	4.920
Stella	ST	16	0.006	2	0.400	10	4.000
Trasimeno	TR	34	0.004	5	0.408	13	2.806
All samples		153	0.008	10	0.726	22	5.455

**Table 2:** Estimates of mtDNA COI genetic diversity indexes for all basins here analyzed (N = number of samples; π = nucleotide diversity; Nh = number of haplotypes; Hd = haplotype diversity; S = number of polymorphic sites; k = Tajima’s average number of nucleotide differences).

Basin	Basin Code	N	π	Nh	Hd	S	k
Bolsena	BO	6	0.000	1	0.000	0	0.000
Candia	CA	8	0.001	3	0.464	3	0.750
Massaciuccoli	MA	14	0.003	3	0.560	5	1.516
Posta Fibreno	PF	6	0.002	2	0.600	2	1.200
Stella	ST	4	0.004	4	1.000	4	2.167
Trasimeno	TR	15	0.003	4	0.619	6	1.695
All samples		53	0.002	6	0.549	10	1.322

## Data Availability

The data presented in this study will be publicly available from the corresponding authors upon publication.

## Supplementary Materials

The following supporting information can be downloaded at: https://www.mdpi.com/article/10.3390/biology12020313/s1. Table S1: List of 153 *P. clarkii* samples here analyzed and their mitochondrial control region and cytochrome c oxidase subunit I (COI) gene haplotypes; Table S2: Haplotype frequency (%) distribution for each basin based on 153 mtDNA control regions and 53 COI sequences; Table S3: Summary of mtDNA haplotypes (control region and COI) from this study observed in other countries; Table S4: Frequencies (%) of unique and shared haplotypes of mtDNA control region and COI sequences among all *P. clarkii* analyzed in this study and available from GenBank; Table S5: Concatenated mitochondrial cytochrome oxidase I (COI) gene and control region (CR) haplotypes.
